# Lymphocyte Cell-Cycle Inhibition by HLA-G Is Mediated by Phosphatase SHP-2 and Acts on the mTOR Pathway

**DOI:** 10.1371/journal.pone.0022776

**Published:** 2011-08-24

**Authors:** Farah Ketroussi, Massimo Giuliani, Rajia Bahri, Bruno Azzarone, Bernard Charpentier, Antoine Durrbach

**Affiliations:** 1 INSERM U1014, Villejuif, France; 2 Département de Néphrologie, Université Paris XI, Hôpital du Kremlin-Bicêtre, IFRNT, Le Kremlin-Bicêtre, France; Massachusetts General Hospital, United States of America

## Abstract

Human leukocyte antigen G (HLA-G) is involved in regulating T-cell responses through its interaction with inhibitory receptors belonging to the immunoglobulin-like transcript family (ILT). In this context, we investigated the pathways involved in the control of cell-cycle entry of T cells following HLA-G interaction with its inhibitory receptor. We show that HLA-G acts through its interaction with the LILRB1 receptor expressed on T lymphocytes. Both HLA-G and LILRB1 antibodies block the inhibitory effect of HLA-G and restore T-cell proliferation. The interaction of HLA-G with T lymphocytes is associated with phosphorylation of SHP-2 phosphatase, but not SHP-1. In addition, in activated T cells, their incubation with HLA-G is not associated with a decrease in the TCR or CD28 downstream pathways, but is associated with dephosphorylation of the mTOR molecule and p70S6K. In contrast, Akt, which acts upstream of mTOR, is not affected by HLA-G. The inhibition of SHP-2 by NSC-87877(5 µM), a chemical inhibitor of SHP-2, or the use of siRNA, abrogates dephosphorylation of mTOR and impairs the overexpression of p27^kip^ in the presence of HLA-G. Together, these results indicate that HLA-G is associated with activation of phosphatase SHP-2, which inhibits the mTOR pathway and favors the inhibition of the cell-cycle entry of human-activated T cells.

## Introduction

Human leukocyte antigen G (HLA-G) participates in graft tolerance and inhibits proliferation of allogenic T cells. HLA-G is a non-classical MHC class I molecule with a limited polymorphism and has restricted tissue distribution: it is only expressed in physiological conditions in medullary thymic epithelial cells [1], in the cornea [2], and in extra-embryonic tissues. During pregnancy, HLA-G is expressed on the cytotrophoblast and is believed to inhibit maternal NK cell cytotoxicity, thus allowing development of the embryo [3]. During human-organ transplantation, HLA-G expression correlates with improved allograft acceptance [4]–[7] in cardiac, lung, combined liver–kidney, or kidney transplantations.

*In vitro*, HLA-G modulates the function of several immune effectors: it acts on natural killer cells (NK) by inhibiting their cytotoxicity [8]–[10] and their transendothelial-migration properties [11]. HLA-G also inhibits antigen-specific CD8^+^ T cell cytolytic function [12], [13], interacts with CD4 T cells and dendritic cells (DC), which are involved in the initiation of the CD4-cell activation cascade during the alloimmune response and favor the expansion of regulatory T cells [14]. HLA-G suppresses CD4^+^ T cell proliferation in response to allogeneic stimulation [15]–[17] and promotes (Th2)-type responses. It also inhibits DC maturation [18], [19], thus increasing allogeneic skin-graft survival.

The inhibitory mechanism of HLA-G on activated T cells remains controversial. HLA-G has been demonstrated to induced apoptosis of T cells activated by phytohemagglutinin (PHA) [20] and a fraction of PHA-activated CD8 cells through the Fas pathway, leading to activation of caspases [13], [21]. In contrast, we have observed that T cells activated through engagement of their T-cell receptor (TCR) are inhibited by HLA-G, but do not undergo apoptosis. This process is associated with inhibition of cell-cycle entry.

HLA-G receptors on immune cells belong to the killer immunoglobulin-like receptor (KIR) [22] and immunoglobulin-like transcript (ILT) families [23], [24]. LILRB1 is mostly expressed on NK cells, and is also expressed intracellularly by most CD4 and CD8 T cells, and by a significant fraction at their surface [25], whereas LILRB4 is expressed on dendritic cells. This suggests that HLA-G can regulate their functions through its interaction with these receptors. *In vitro*, we have shown that the inhibitory properties of HLA-G depend on its interaction with LILRB1 at the cell surface of lymphocytes whereas the regulatory effect of HLA-G on DC is mediated by LILRB2 and LILRB1. CD85j (LILRB1) is a 110-kDa surface glycoprotein detected on the surface of NK and T-cell subsets, B cells, dendritic cells, and monocytes [1], [2]. CD85j includes four Ig-like C2 domains in its extracellular region, which interact with the alpha domain of HLA-G and with UL18, a human cytomegalovirus (HCMV) protein homologous to HLA class I molecules [26], [27]. Its intracellular domain contains four ITIM-like sequences [4] that have been demonstrated in Jurkat cells to interact with phosphatase SHP-1 and thereby inhibit the phosphorylation of MAP kinases [28]. In addition, crosslinking of LILRB1 is associated with dephosphorylation of several proteins activated downstream of the FcγR-dependant pathway in monocytes, and leads to the hypothesis that a phosphatase is activated to inhibit T-cell activation [29]. In DC, HLA-G has been demonstrated to activate SHP-2 through its interaction with LILRB4 and thereby limit the activation of DC.

In this report, we have analyzed the pathways affected during the incubation of HLA-G with activated T cells. We show that HLA-G, by interacting with CD85j, does not activate SHP-1 but activates another phosphatase, SHP-2, and inhibits the mTOR pathway.

## Materials and Methods

### Cell culture

Peripheral blood leukocytes were isolated from blood-bank leukophoresis packs obtained from healthy volunteers (EFS, Saint Louis Hospital, France). After Ficoll-Isopaque density (Eurobio) gradient centrifugation, adherent cells were removed by incubating cells on plastic dishes. T cells were stimulated for 3 days with 0.25 µg/ml OKT3 and 100 U/ml IL-2 in RPMI 1640 medium with 10% decomplemented fetal bovine serum (FBS).

### Proliferation assays

Proliferation responses were evaluated by culturing 10^5^ T lymphocytes in the presence of 5×10^4^ M8 cells pretreated with mitomycin in 0.150 ml of complete medium, or by directly activating T lymphocytes with OKT3 and IL-2, in 96-well flat-bottom plates.

Cultures were pulsed with 1 µCi [3H]thymidine (Amersham, San Francisco, CA) on day 3 or 5, and harvested 18 h later. The dry filters were counted in a beta counter with scintillation fluid.

Results are given as mean values of triplicate cultures.

### Cell lines

The HLA class I-positive M8 melanoma cell line was kindly provided by F. Jotereau (INSERM U211, Nantes, France) and was transfected with full-length HLA-G5 cDNA (without the transmembrane sequence) (M8-HLA-G5) subcloned in vector pcDNA (Invitrogen, San Diego, CA). Stable cell lines were selected with geneticin 418 (0.5 mg/ml) (Invitrogen, San Diego, CA) in complete medium as previously described [8], [30]. The M8-HLA-G5 transfectant and control cells (M8 melanoma transfected with the vector alone) (M8-pcDNA) were used, respectively, as positive and negative controls [31]. HELA cells were purchased from ATCC.

### Reagents and antibodies

Antibodies against ILT-2 (sc-20065), SHP-2 (sc-280), p27^kip^ (sc-527), p-p70S6 kinase (Thr421/Ser424)-R and HLA-G (4H84 mAb) (Figure S1) were purchased from Santa Cruz Biotechnology. Phospho-mTOR (Ser2448), phospho-SHP-2(Tyr542), phosphor-p44/42MAPK (ERK1/2) (Thr2002/Tyr204) (197G2), and phospho-Akt (Ser473) were obtained from Cell Signaling. Anti-actin (A2668) was obtained from Sigma Aldrich, and anti-human CD247 (CD3ξchain) was purchased from BD Pharmingen.

The monoclonal antibody to HLA-G (87G) was purchased from EXBIO (Czech Republic).

SHP-1 was obtained from Exalpha Biologicals, and phospho-SHP-1 (phosphor Y536) from Abcam.

For western-blot analysis, immunoreactive proteins were visualized with HRP-coupled goat anti-mouse IgG (Amersham Biosciences, Buckinghamshire, UK) or HRP-conjugated sheep anti-rabbit IgG (Biotest, BUC, France) using an ECL detection system (ECL kit, Amersham Biosciences, Buckinghamshire, UK). Anti-CD3 mAb (OKT3) was obtained from Orthoclone. For immunofluorescence, phycoerythrine-coupled donkey anti-mouse IgG and fluorescein-coupled goat anti-rabbit IgG were purchased from Jackson ImmunoResearch.

### HLA-G5 production by M8 melanoma cell lines and immune-purification of HLA-G with magnetic beads

Recombinant HLA-G5 protein was produced in a HLA class I-positive M8 melanoma cell line transfected with HLA-G5 cDNA. Supernatant from M8-HLA-G5 cells was used as a source of HLA-G5 and was filtered (0.22 µm) before HLA-G5 could be immunopurified with magnetic beads. The beads were coated covalently on their surfaces with anti-mouse IgG (Bio-Adembeads goat anti-mouse antibodies, Ademtech, France), and then incubated with anti-HLA-G antibody (4H84) [32]. After washing in RPMI, the 4H84 coated beads were incubated overnight at 4°C with M8-HLA-G5 supernatant. The beads were then washed and suspended in culture medium. Control beads were incubated with supernatant of M8 melanoma transfected with the pcDNA empty vector.

### Biochemistry and western blot analysis

Cells were activated for 3 days with OKT3 and IL-2, and incubated in the absence or presence of 30 µl of HLA-G5-beads. Then, cells were washed three times with ice-cold PBS and lysed with buffer containing 1% Triton X-100, 50 mM Tris (pH = 7.4), 150 Mm NaCl, 2 mM EDTA, 1% Nonidet-P40, Benzonase 0.5 U/ml, 1 mM Orthovanadate, and 1% protease inhibitors 25× (Complete; Boehringer Mannheim). the cells were then boiled in Laemmli buffer 2× with 10% β2-mercaptoethanol.

Total protein was extracted and quantified using the BCA protein assay kit (Pierce). Proteins were then resolved on 7.5% SDS–PAGE and transferred onto polyvinylidene difluoride (PVDF) membranes.

After blocking non-specific binding with 5% bovine serum albumin (BSA) in PBS-0.1%Tween for 1 h at room temperature, the membranes were immunoblotted using primary antibodies. Immunoreactive proteins were visualized with horseradish peroxidase-coupled HRP-conjugated goat anti-mouse IgG or HRP-conjugated sheep anti-rabbit IgG, and enhanced using a chemiluminescence detection system.

### Immuno-precipitation and immunoblotting

Cells were stimulated with OKT3 and IL-2 for 3 days, and incubated in the absence or presence of 30 µl of HLA-G5-beads for 15 min, 30 min, and 1 h at 37°C. Afterwards, the cells were washed and lysed at 4°C with a buffer solution containing 1% TritonX100, 5 mM EDTA, 100 mM NaCl, 50 mM Tris–HCl (pH = 7.4), 1 mM orthovanadate, 1 mM PMSF (phenylmethylsulphonyl fluoride), and 1% protease inhibitors 25× (Complete; Boehringer Mannheim) for 10 min. The lysates were then centrifuged (2000 rpm) and the supernatant was incubated overnight at 4°C with specific antibodies, and then with magnetic beads coated with goat anti-mouse IgG for 2 h at 4°C. After washing with lysed buffer, the protein–antibody-bead complexes were boiled in a Laemmli buffer 2×, resolved on 8% SDS–PAGE, and were analyzed using western-blot methodology.

### Inhibition of SHP-2

In order to interfere with the function of SHP-2, stimulated T cells were pre-treated with a specific chemical inhibitor, NSC-87877 (5 µM), for 3 h [33]. To inactivate the SHP-2 gene, cells were transfected with anti-SHP-2-siRNA (75 ng) or control siRNA (75 ng) (Dharmacon) with HIPerfect Transfection reagent (Qiagen). Cells were transfected with 75 ng of siRNA and HIPerfect for 48 h. T cells were stimulated with OKT3 and IL2 for 24 h in the absence or presence of 30 µl of HLA-G5-beads for 1 h. Total protein extracts then underwent western-blot analysis.

### Immunofluorescence

Cells were activated for 3 days, and incubated with HLA-G5-beads for 15 min, 30 min, 1 h, and 2 h. Afterwards, cells were washed twice with ice-cold PBS and fixed with 3% paraformaldehyde (PFA) in PBS for 30 min at 4°C. Cells were then incubated with NH_4_Cl 100 mM in PBS for 10 min. Cells were permeabilyzed with PBS containing 0.05% Triton X100 for 5 min and stained with primary antibodies for 1 h, followed by secondary antibodies conjugated with a specific fluorochrome for 1 h at room temperature.

For primary antibodies, anti-SHP-2 was diluted at 1∶100 and anti-ILT-2 was diluted at 1∶25. Nuclei were stained with DAPI (1∶5000). The secondary antibodies used were goat anti-rabbit IgG coupled with FITC (1∶100; Jackson Immuno Research), and donkey anti-mouse IgG coupled with PE (1∶25; Jackson Immuno Research).

## Results

### LILRB1 is required for the inhibitory effect of HLA-G on T lymphocytes

**Figure 1:**
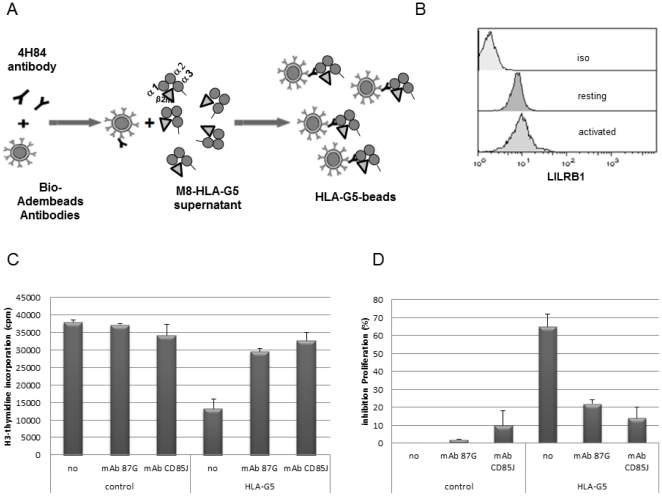
Inhibition of proliferation of T cells stimulated with OKT3/IL2 in the presence or absence of HLA-G, and with or without HLA-G- or LILRB1-blocking antibodies. **A**: Scheme of the interaction between 4H84 coated beads with HLA-G5. **B**: Cell surface expression of LILRB1 on lymphocytes analyzed by FASC using the CD85J mAb. The isotype control mAb was tested on resting T cells (Iso). LILRB1 is expressed both on resting and activated T cells. **C**: Thymidine incorporation into T cells stimulated with OKT3/IL2 and incubated or not with HLA-G- or LILRB1-blocking antibodies or the HLA-G-blocking antibody (87G) **D**: The inhibition of T-cell proliferation associated with HLA-G5 was dramatically reduced when the HLA-G-inhibitory mAb(87G)- or LILRB1-blocking antibody were added to T cells in the presence of HLA-G.

**Figure 2:**
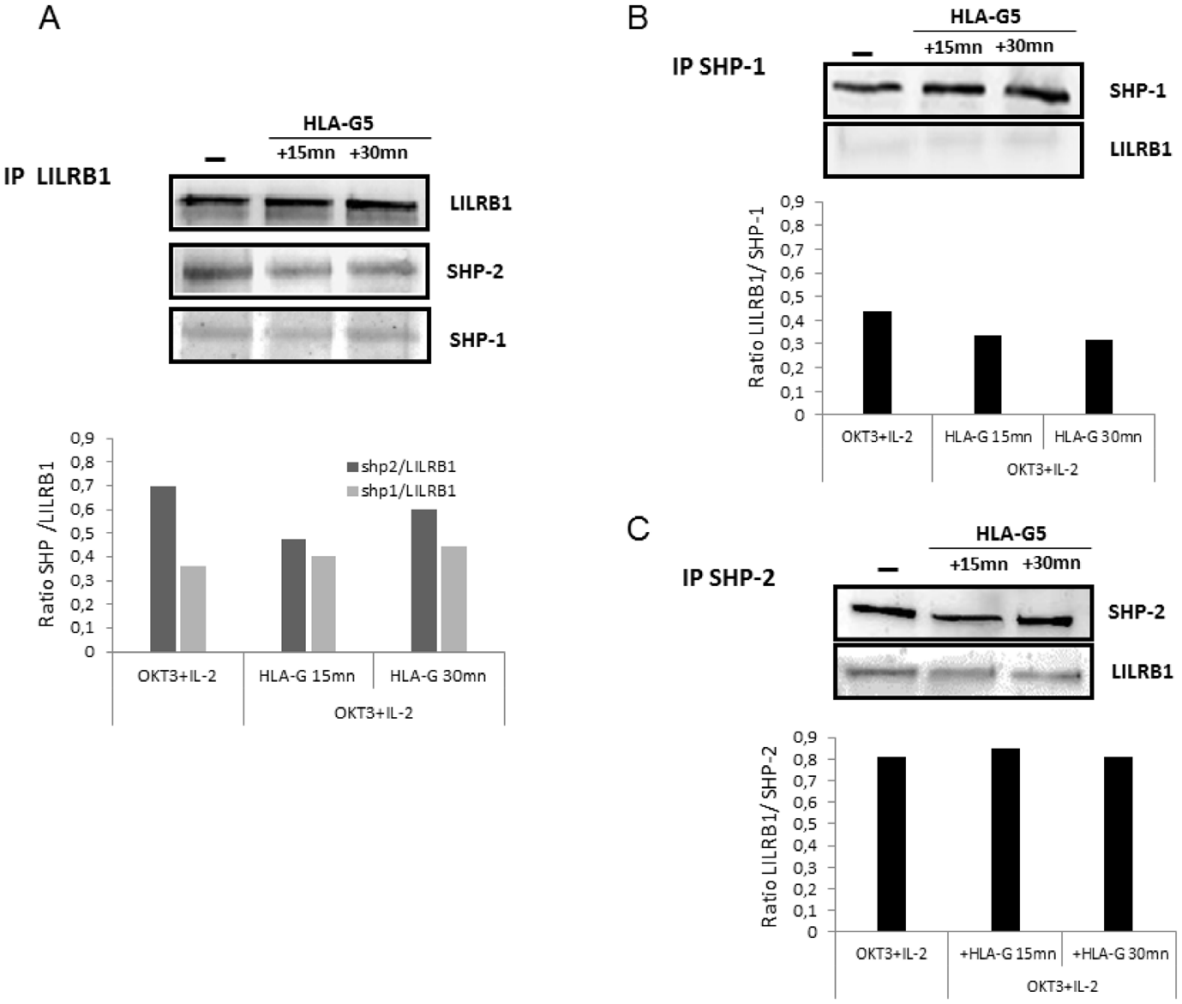
Immuno-precipitation of ILT-2, SHP-1, or SHP-2 in T cells in the presence or absence of HLA-G. T cells were stimulated with OKT3/IL2, and were incubated with HLA-G5-coated beads for 0, 15 or 30 min. After cell lysis, LILRB1, SHP-1, or SHP-2 were immuno-purified and the co-precipitated molecules were submitted to gel electrophoresis and then transferred to a PVDF membrane. SHP-1, SHP-2, and LILRB1 were detected by western-blot analysis. **A**: Immunoprecipitation of LILRB1. LILRB1, SHP-1 and SHP-2 were detected by western blot. The ratio of SHP-1 or SHP-2/LILRB1 have been determined by densitometry. **B**: Immunoprecipitation of SHP-1. LILRB1 and SHP-1 were detected by western blot. The ratio of LILRB1/SHP-1 has been determined by densitometry. C Immunoprecipitation of SHP-2. LILRB1 and SHP-2 were detected by western blot. The ratio of LILRB1/SHP-2 has been determined by densitometry.

In the presence of soluble HLA-G, proliferation of activated T cells is inhibited. This inhibition is dramatically reduced in the presence of the HLA-G blocking antibody (87G) and/or the presence of the anti-LILRB1 antibody, indicating a requirement for LILRB1 in the inhibitory function of HLA-G (Figure 1A, B). To determine which phosphatase is associated with LILRB1, LILRB1 was immune-precipitated and submitted to gel electrophoresis. Co-precipitated SHP-1 or SHP-2 phosphatases were detected by western-blot analyses. In human-activated T lymphocytes, SHP-2, and to a less extend SHP-1, was associated with LILRB1 (Figure 2). This was confirmed by immuno-precipitating SHP1 or SHP-2, and subsequently detecting LILRB1 by western blotting. SHP-2 and to a less extend SHP-1 were associated with LILRB1 (Figure 2).

**Figure 3:**
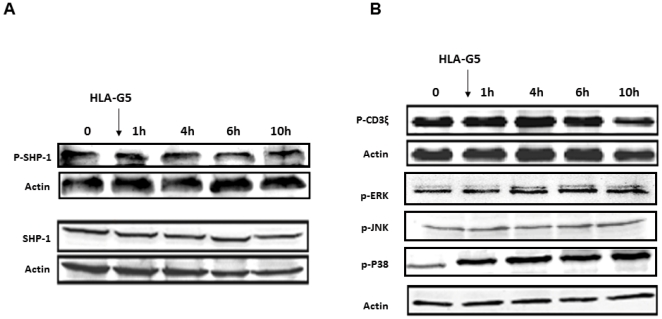
Determination by Western Blot of the expression of SHP-1, p-SHP-1 (A), and p-CD3ξ, p-ERK, p-JNK, and p-P38 (B) following incubation of activated T cells with OKT3/IL2 in the presence of HLA-G5-coated beads.

**Figure 4:**
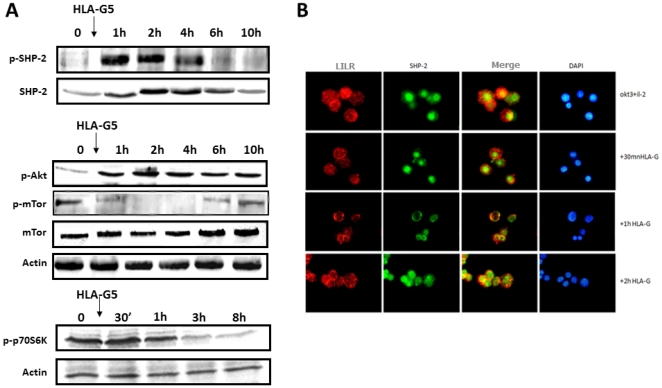
A: Time-course modification of pSHP-2, p-Akt, p-mTOR, and p70S6K on activated T cells incubated with HLA-G5-coated beads. T lymphocytes were activated before incubation with OKT3/IL-2 for 72 h. In the presence of HLA-G, p-mTOR and p70S6K were transiently dephosphorylated, but Akt was not. This result correlates with SHP-2 phosphorylation. **B**: Intracellular distribution of ILT-2 and SHP-2 in the presence of HLA-G as assessed by immunofluorescence. In stimulated T cells incubated with HLA-G5-coated beads, SHP-2 was redistributed intracellularly and translocated from the nucleus to the cytoplasm, whereas it was only partially redistributed after 1 h with LILRB1.

**Figure 5:**
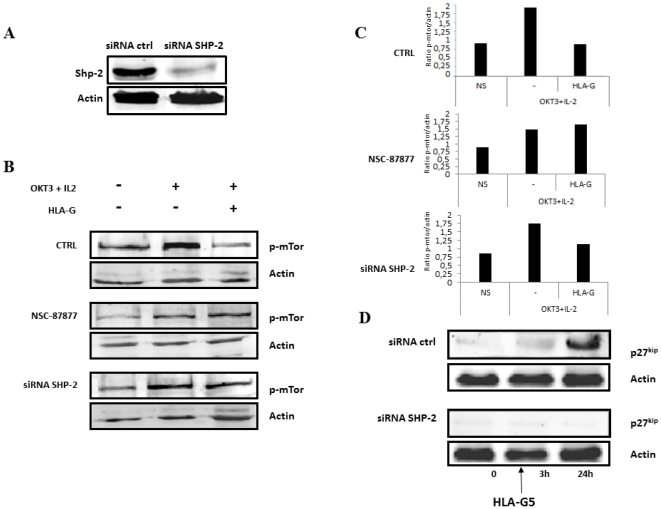
A: Transcriptional inhibition of SHP-2 with siRNA against SHP-2. Activated T cells were transfected with siRNA against SHP or a siRNA control, and submitted 48 h later to gel electrophoresis. SHP-2 was determined by western blotting. siRNA transfected against SHP-2 resulted in a dramatic reduction of SHP-2. **B**: Transcriptional and functional inhibition of SHP-2 with siRNA against SHP-2 or with the specific inhibitor of SHP-2 (NSC-87877) in T cells in the presence of HLA-G5-coated beads. In activated T lymphocytes transfected with siRNA–SHP-2 or incubated with NSC-87877, incubation with HLAG5-coated beads was not associated with mTOR dephosphorylation. **C**: The ratio of p-mTOR/actin has been determined by densitometry **D**: Effect of HLA-G5 on p27^kip^ after inhibition of SHP-2. Stimulated T cells were transfected with siRNA–SHP-2 or control siRNA (siRNA ctrl) and incubated with HLA-G5-coated beads for 24 h. In T lymphocytes transfected with siRNA against SHP-2 (siRNA-SHP-2), p27^kip^ did not appear following incubation with HLA-G5, whereas it was observed in T cells transfected with siRNA–control.

After incubation with HLA-G, the amount of SHP-2 that co-precipitated with LILRB1 was slightly decreased. SHP-1 remained not associated with LILRB1. We also searched for the phosphorylated status of SHP-1 and SHP-2. We found that the incubation of activated T cells with HLA-G was not correlated with the rapid phosphorylation of SHP-1 (Figure 3A), but was with SHP-2 (Figure 4A). The phosphorylation of SHP-2 was observed at 1 and 2 h after incubating the cells with HLA-G and then decreased after 4 h. In addition, the amount of SHP-2 was increased as shown Figure 4A, but was delayed compared to its phosphorylation. In contrast, the amount of SHP-1 was not modified. By immunofluorescence, the distribution of SHP-2 was rapidly modified following the interaction of HLA-G with its receptor. SHP-2, which was distributed in the cytoplasm and also in the nucleus at a steady state, will thus be relocated intracellularly and partially co-distributed with LILRB1 when they have been incubated with HLA-G (Figure 5).

### HLA-G is associated with inhibition of the m-TOR pathway, but not the TCR-dependent pathway

To investigate which pathways of activated T cells were affected by phosphatase SHP-2, we first tested whether the TCR-dependent pathway could be modified by incubating T cells with HLA-G. In activated T cells, the phosphorylated zeta chains of the CD3 complex remained phosphorylated (Figure S2). Both ERK and JNK, which are phosphorylated following TCR activation, were not modified and remained phosphorylated. Only p38 phosphorylation was increased after 1 h of HLA-G incubation (Figure 3B).

Because the proliferation of T cells depends on IL2, which in turn activates the PI3K/Akt/mTOR pathway (the third signal for the activation of T cells), we determined the status of these proteins (Figure 4A). In activated T cells, both Akt and mTOR were phosphorylated. In the presence of HLA-G, phospho-Akt was increased but phospho-mTOR was dramatically reduced, whereas the quantity of mTOR was not modified: this suggests that HLA-G impairs the phosphorylated status of mTOR. Moreover, p70-S6K, which acts downstream of mTOR, was also dephosphorylated in a time-dependent manner, indicating that mTOR was not functional any longer.

### SHP-2 control of the phosphorylated status of mTOR

To demonstrate the interaction between the activation of SHP-2 and inhibition of the mTOR pathway, the function of SHP-2 was inhibited, firstly, by using a specific inhibitor of SHP-2 (NSC-87877) and, secondly, by using siRNA against SHP-2. Transfection of T cells with siRNA against SHP-2 was associated with a dramatic decrease of SHP-2 in cells determined by western-blot analysis as compared to cells transfected with controlled siRNA (Figure 4B). As expected, in untreated cells, the incubation of activated T cells with HLA-G was associated with a significant reduction of phospho-mTOR. In contrast, in activated T cells incubated with NSC-87877 or transfected with the siRNA against SHP-2, incubation with HLA-G was not associated with decreased phospho-mTOR (Figure 4C). This indicates that HLA-G induces mTOR dephosphorylation through SHP-2 activation. In contrast, in cells transfected with control si-RNA, the incubation of HLA-G led to dephosphorylation of m-TOR (data not shown).

To determine whether the regulation of the mTOR pathway by SHP-2 is important for the control of cell-cycle entry induced by HLA-G, activated T lymphocytes were transfected with siRNA against SHP-2 prior to incubation with HLA-G (figure 4D). Following incubation of the cells with HLA-G, the reappearance of p27^kip^ was determined by western-blot analysis. In cells transfected with siRNA against SHP-2, p27^kip^ did reappear after incubation of cells with HLA-G whereas, in cells transfected with control siRNA, it was not expressed.

## Discussion

Our results indicate, for the first time, that the regulatory functions of HLA-G on T-cell proliferation is associated with the activation of SHP-2, which induces mTOR inhibition.

The function of HLA-G requires interaction of its α3 domain with LILRB1, and this can be inhibited by using blocking antibodies against either HLA-G or LILRB1. LILRB1 contains, in its intracellular domain, four ITIM motifs that participate in the transmission of the negative signal. Following its phosphorylation, LILRB1 has been demonstrated to interact with phosphatase SHP-1 through its SH2 domain. In activated lymphocytes, we found no specific interaction between LILRB1 and SHP-1 in T cells incubated or not with HLA-G. However, we observed that LILRB1 was associated with ubiquitous phosphatase SHP-2 in activated T cells, but not in resting lymphocytes (data not shown). Our results suggest that the activation of T cells is associated with the direct or indirect modification of SHP-2 and/or LILRB1, thus participating in SHP-2 activation. This is in accordance with the results published by Liang et al., which show that in dendritic cells overexpressing LILRB2, another member of the ILT family, HLA-G correlated with the association of SHP1 and, to a lesser extent, SHP-2 with LILRB2 [34]. However, only SHP-2 downregulation by shRNA was associated with the inhibition of the regulatory function of HLA-G, indicating that SHP-2 is required for signal transmission in dendritic cells downstream of LILRB4.

After HLA-G interaction, SHP-2 becomes rapidly phosphorylated. In addition, the total amount of SHP-2 was increased with some delay compared to its phosphorylation. The phosphorylation of SHP-2 correlates with a redistribution of SHP-2 into the cytoplasm of the cell. In contrast to the transmembrane phosphatase CD45, activation of SHP1 or SHP-2, the two phosphatases without no transmembrane domains, can impair the first signal of activation [35], [36]. In human T cells activated with either OKT3/CD28mAb or OKT3/IL2, several lines of evidence suggest that HLA-G does not interfere with the first activation signal of T cells. Activated-T cells, in the presence of HLA-G, first exhibit a phenotype of the activated cells with regard to their size and granularity (39), and then, secondly, the expression of surface markers (CD45R0^+^, CD62L^−^, CD45RA^−^). In addition, we observed that, in the presence of HLA-G, the CD3ζ chain, MAP kinase, JNK, and p38, which all act downstream of TCR and CD28, are not modified following incubation with HLA-G and remain phosphorylated. In contrast, we have observed that the third signal of activation, which involves the Akt/mTOR pathway downstream of the IL2 receptor, was modulated. After HLA-G binding, the mTOR/raptor molecule and p70S6K, which act downstream of mTOR, appear to become rapidly dephosphorylated, whereas Akt, which acts upstream of mTOR, is not downregulated. These results are in agreement with previous results, indicating that, in SHP-2, KO only affects the activation status of mTOR but not Akt in fibroblasts stimulated with IGF1 (40). IGF1 interacts with its receptor to turn on the Akt/mTOR pathway by activating Jak1/3 molecules, as IL2 does in T cells. By using SHP-2 KO mice, Zito et al. have demonstrated that, in the absence of SHP-2, a higher phosphorylation status of mTOR was observed. However, the mechanism that regulates SHP-2 activation in fibroblasts remains unknown.

By using a specific inhibitor of SHP-2, NSC-87877, or by inhibiting SHP-2 with siRNA, we have shown that mTOR is phosphorylated regardless of the presence of HLA-G: this indicates that, as for fibroblasts, SHP-2 regulates the mTOR pathway. We also found that SHP-2 acts upstream and does not reduce the phosphorylated status of Akt. In addition, by using siRNA, we have demonstrated that SHP-2 participates in the inhibition of cell-cycle entry and is associated with the accumulation of protein p27^kip^. In addition, based on these results, Morandi et al. have observed in T cells, that the chemical inhibitor of SHP-2 restored the altered expression of cell-surface expression of chemokine receptor CXCR5 when it was induced by HLA-G [37]. Altogether, these data indicate that SHP-2 has the ability to control the cytokine/growth-factor pathway by acting downstream of Akt and inhibiting the active form of mTOR. Whether hyperphosphorylation of Akt plays a role in this sequence needs to be determined.

## Supporting Information

Figure S1 **Determination of the expression of cDNA encoding HLA-G transfected cells with the 4H84 mAb.** A: HELA cells transitly transfected with the cDNA encoding HLA-G5 were fixed with paraformaldehyde 3% and then permeabelized with saponin 0.01%. They were subsequently incubated with FITC conjugated 4H84 mAb (Green) and DAPI. B: Melanoma cell line (M8) stably transfected with the cDNA encoding HLA-G1 were incubated with the biotinylated MEM-G9 mAb and then FITC-streptavidin (left panel) (dark gray) or with the FITC conjugated 4H84 mAb (right panel) (dark gray) and analysed by FASC. Isotype control appears in light gray.
(TIF)

Figure S2 **A: Determination by Western Blot of the expression of p-CD3γ and actin following incubation of activated T cells with OKT3/IL2 in the presence of HLA-G5-coated beads.** B: The ration of p-CD3γ/Actin has been determined by densitometry.
(TIF)
