# Supplementary figures and images for: Lymphocyte Cell-Cycle Inhibition by HLA-G Is Mediated by Phosphatase SHP-2 and Acts on the mTOR Pathway
Source: PLoS One. 2011 Aug 24;6(8):e22776. doi: 10.1371/journal.pone.0022776 (PMC3160837; doi:10.1371/journal.pone.0022776)

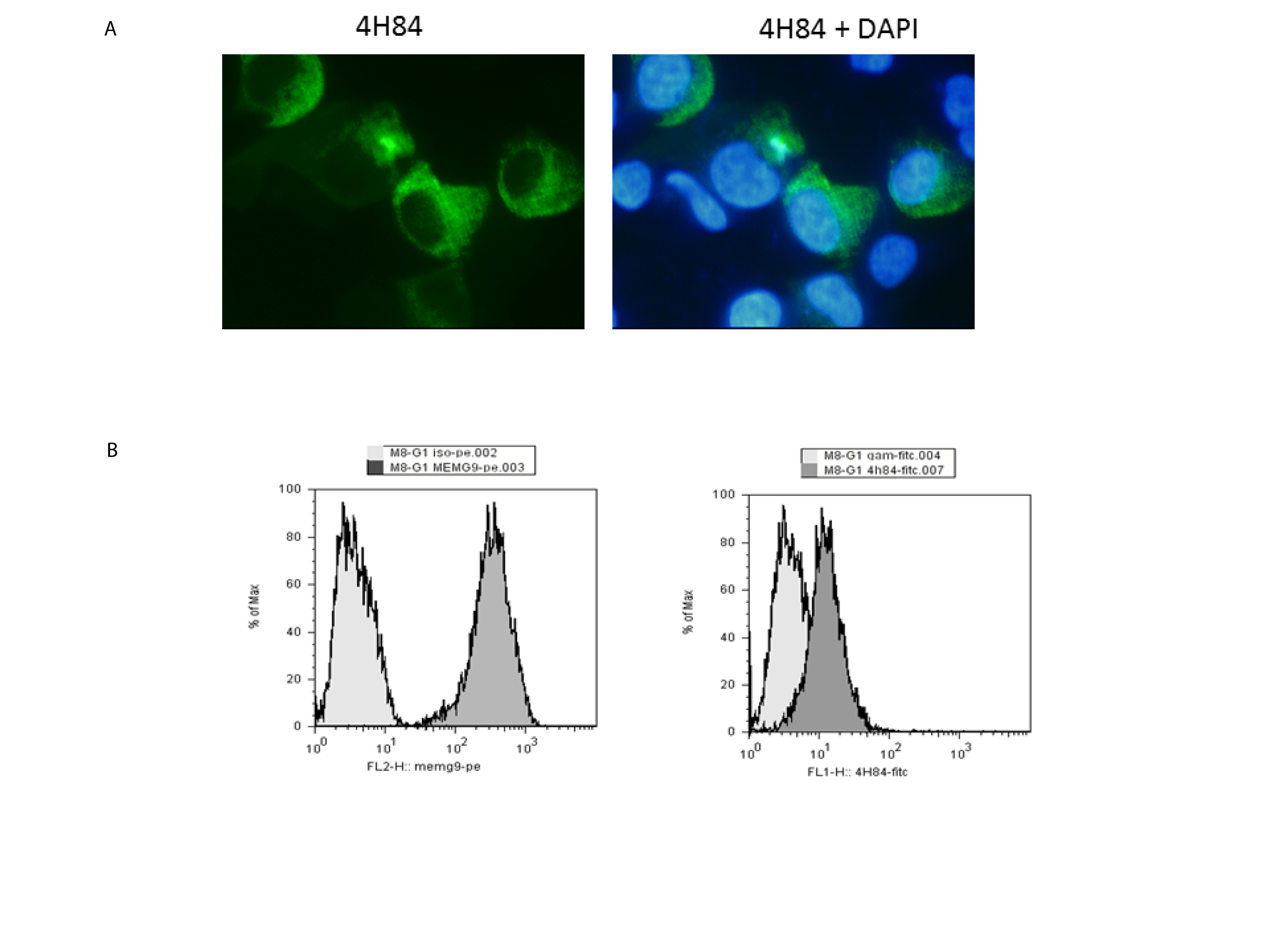

Supplement: Figure S1 — Determination of the expression of cDNA encoding HLA-G transfected cells with the 4H84 mAb. A: HELA cells transitly transfected with the cDNA encoding HLA-G5 were fixed with paraformaldehyde 3% and then permeabelized with saponin 0.01%. They were subsequently incubated with FITC conjugated 4H84 mAb (Green) and DAPI. B: Melanoma cell line (M8) stably transfected with the cDNA encoding HLA-G1 were incubated with the biotinylated MEM-G9 mAb and then FITC-streptavidin (left panel) (dark gray) or with the FITC conjugated 4H84 mAb (right panel) (dark gray) and analysed by FASC. Isotype control appears in light gray. (TIF) [file pone.0022776.s001.tif]

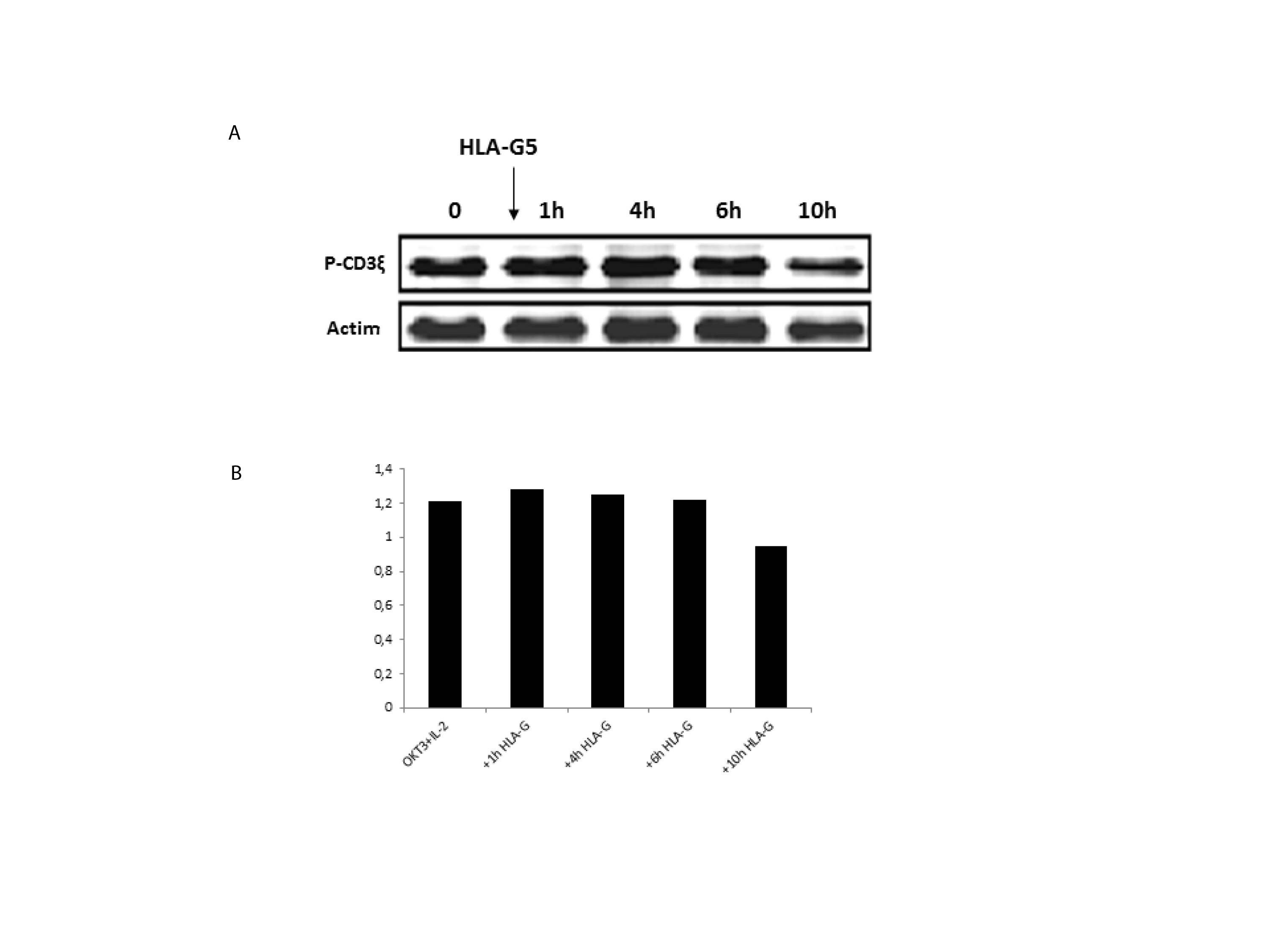

Supplement: Figure S2 — A: Determination by Western Blot of the expression of p-CD3γ and actin following incubation of activated T cells with OKT3/IL2 in the presence of HLA-G5-coated beads. B: The ration of p-CD3γ/Actin has been determined by densitometry. (TIF) [file pone.0022776.s002.tif]
